# Exploring the role of curcumin in mitigating oxidative stress to alleviate lipid metabolism disorders

**DOI:** 10.3389/fphar.2025.1517174

**Published:** 2025-01-30

**Authors:** Maojun Cheng, Fang Ding, Liyang Li, Changmao Dai, Xiaolan Sun, Jia Xu, Feier Chen, Mingxiu Li, Xueping Li

**Affiliations:** ^1^ School of Clinical Medicine, Chengdu University of Traditional Chinese Medicine, Chengdu, Sichuan, China; ^2^ Hosptial of Chengdu University of Traditional Chinese Medicine, Chengdu, Sichuan, China; ^3^ Sichuan No. 2 Hosptial of Traditional Chinese Medicine, Chengdu, Sichuan, China

**Keywords:** curcumin, oxidative stress, lipid metabolism disorders, hyperlipidemia, NAFLD, atherosclerosis, obesity, diabetes

## Abstract

Lipid metabolism plays a crucial role in maintaining homeostasis and overall health, as lipids are essential molecules involved in bioenergetic processes. An increasing body of research indicates that disorders of lipid metabolism can contribute to the development and progression of various diseases, including hyperlipidemia, obesity, non-alcoholic fatty liver disease (NAFLD), diabetes mellitus, atherosclerosis, and cancer, potentially leading to poor prognoses. The activation of the oxidative stress pathway disrupts lipid metabolism and induces cellular stress, significantly contributing to metabolic disorders. A well-documented crosstalk and interconnection between these metabolic disorders exists. Consequently, researchers have sought to identify antioxidant-rich substances in readily accessible everyday foods for potential use as complementary therapies. Curcumin, known for its anti-inflammatory and antioxidant properties, has been shown to enhance cellular antioxidant activity, mitigate oxidative stress, and alleviate lipid metabolism disorders by reducing reactive oxygen species (ROS) accumulation. These effects include decreasing fat deposition, increasing fatty acid uptake, and improving insulin sensitivity. A review of the existing literature reveals numerous studies emphasizing the role of curcumin in the prevention and management of metabolic diseases. Curcumin influences metabolic disorders through multiple mechanisms of action, with the oxidative stress pathway playing a central role in various lipid metabolism disorders. Thus, we aimed to elucidate the role of curcumin in various metabolic disorders through a unified mechanism of action, offering new insights into the prevention and treatment of metabolic diseases. Firstly, this article provides a brief overview of the basic pathophysiological processes of oxidative stress and lipid metabolism, as well as the role of oxidative stress in the pathogenesis of lipid metabolism disorders. Notably, the article reviews the role of curcumin in mitigating oxidative stress and in preventing and treating diseases associated with lipid metabolism disorders, including hyperlipidemia, non-alcoholic fatty liver disease (NAFLD), atherosclerosis, obesity, and diabetes, thereby highlighting the therapeutic potential of curcumin in lipid metabolism-related diseases.

## 1 Introduction

Lipid metabolism encompasses the digestion, absorption, synthesis, and breakdown of fats within an organism, facilitated by various enzymes. These processes enable the conversion of fats into essential substances necessary for maintaining normal physiological functions. As a fundamental and intricate biochemical pathway, lipid metabolism is vital for sustaining overall metabolic balance in the human body (Loix et al., 2024). At the cellular level, lipids, proteins, and nucleic acids are key components of cellular membranes and structural complexes. Lipids primarily function in storage and metabolism, while also serving as key signaling molecules in various cell types. Moreover, the regulation of lipid metabolism is essential for maintaining cellular homeostasis (Bian et al., 2021; Corn et al., 2020). Disturbances in lipid metabolism can result in abnormal cellular function, impaired metabolism, inflammatory responses, cellular damage, and potentially cell death. Disorders of lipid metabolism, as key initiating factors, contribute to metabolic diseases such as hyperlipidemia, nonalcoholic fatty liver disease (NAFLD), atherosclerosis, diabetes mellitus, obesity, and other related conditions, which are often interrelated or exhibit crosstalk. Dyslipidemia is closely linked to various cardiovascular and cerebrovascular diseases, while hyperlipidemia significantly increases cardiovascular risk and affects overall health, particularly in conditions such as atherosclerosis, obesity, and type 2 diabetes (Berberich and Hegele, 2022). In 2021, the global prevalence of diabetes was estimated at 6.1%, corresponding to approximately 529.12 million people. China currently has the highest number of diabetic patients worldwide, and the population of pre-diabetics (including those with impaired glucose tolerance and impaired fasting glucose) continues to rise each year (Abel et al., 2024; Xu et al., 2024). Non-alcoholic fatty liver disease (NAFLD), the most prevalent liver disease worldwide, is estimated to affect 38% of the global population. The prevalence of NAFLD has risen by more than 50% over the past 3 decades, driven by changes in diet and urbanization (Wong et al., 2023). The global prevalence of diseases associated with lipid metabolism disorders, along with both direct and indirect mortality rates, continues to rise annually (Zhang et al., 2018).

Abnormalities in lipid metabolism and oxidative stress are interrelated; disorders of lipid metabolism can increase oxidative stress, which, in turn, exacerbates lipid metabolism disorders, thereby creating a vicious cycle. Additionally, increased oxidative stress in adipose tissue can lead to metabolic deterioration, including limited adipose tissue expansion and reduced insulin sensitivity, which further impairs normal lipid metabolism (Okuno et al., 2018). Reactive oxygen species (ROS) play a role in maintaining the balance of cellular metabolism and biochemical reactions under normal physiological conditions. However, excessive ROS accumulation can induce cellular damage, particularly DNA damage, lead to lipid peroxidation, or even result in cell death (DeVallance et al., 2019; Masenga et al., 2023). Disorders of lipid metabolism not only disrupt cellular function and lipid homeostasis, but also significantly influence the development, progression, and prognosis of related diseases. Regarding treatment, the first approach involves non-pharmacological interventions, such as dietary control, weight reduction, regular exercise, and other lifestyle modifications (Lundell et al., 2020). For severe disorders of lipid metabolism, drug interventions, such as statins, are required. If the disease has progressed, appropriate, systematic, and evidence-based pharmacological therapy should be administered alongside lipid regulation (Rong et al., 2022). However, limitations in the effectiveness of drug therapies and their adverse effects have prompted clinical and scientific researchers to explore additional complementary treatments. Due to their proven efficacy and safety, botanical drug and natural medicines have been extensively studied as alternative therapies for regulating lipid metabolism disorders and their associated diseases (Li et al., 2020; Pivari et al., 2019).

The herb turmeric is a curry spice originating from Southeast Asia, whose main bioactive metabolites is curcumin (1,7-bis-(4-hydroxy-3-methoxyphenyl)-hepta-1,6-diene-3,5-dione) (Kotha and Luthria, 2019). Curcumin, exhibits numerous health benefits and pharmacological properties (Yin et al., 2022). Numerous experimental studies from diverse methodologies have demonstrated curcumin’s anti-inflammatory, antioxidant, and lipid-modulating properties (Helli et al., 2021; Lopresti et al., 2021; Pivari et al., 2022), improvement of insulin resistance (Thota et al., 2020), inhibition of tumor cell proliferation and metastasis (Zhang Z. et al., 2016), among other effects. These positive pharmacological effects highlight curcumin’s considerable potential in treating related diseases. Curcumin works by modulating key oxidative stress pathways to enhance cellular antioxidant activity while attenuating oxidative stress effects (Ashrafizadeh et al., 2020; Rahban et al., 2020). This makes it an important candidate for the treatment of several oxidative stress-related diseases.

**FIGURE 1:**
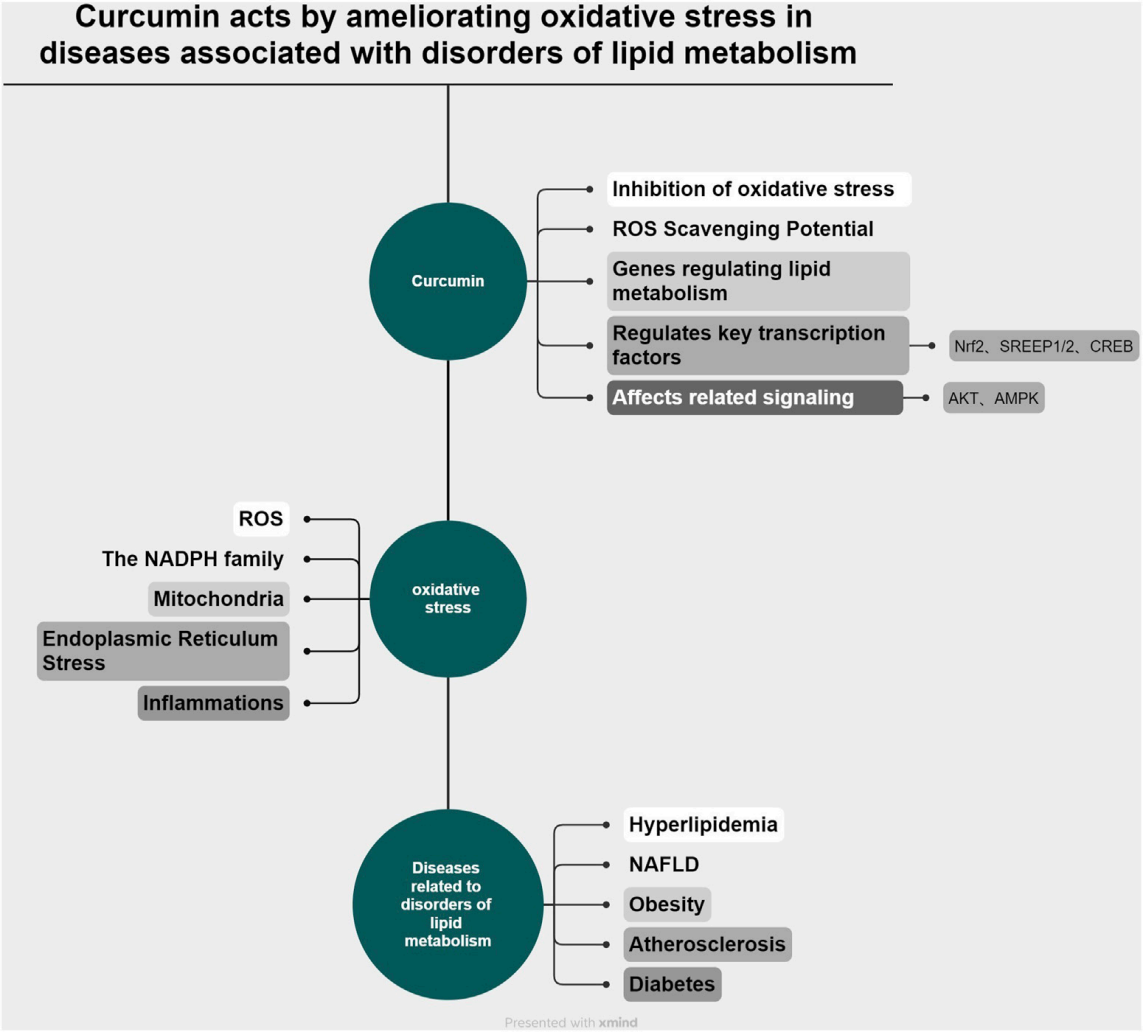
(by Xmimd): Schematic representation of the relationship among curcumin, oxidative stress, and disorders associated with lipid metabolism.

In this review, we first examine the interconnected relationship between lipid metabolism, curcumin, and oxidative stress. Then, we focus on the therapeutic effects of curcumin on hyperlipidemia, nonalcoholic fatty liver disease, atherosclerosis, obesity, and diabetes mellitus associated with lipid metabolism disorders, through modulation of oxidative stress pathways, highlighting both its effectiveness and limitations to inform future research. In addition, the primary methods and strategies for enhancing the bioavailability of curcumin are reviewed. In conclusion, additional research avenues and clinical therapeutic directions are suggested for the treatment of diseases associated with lipid metabolism disorders using curcumin. (The relationship between curcumin, oxidative stress, and diseases associated with disorders of lipid metabolism is shown in Figure 1).

## 2 Methodology

For a more comprehensive understanding of diseases associated with curcumin, oxidative stress, and lipid metabolism disorders, as well as their interrelationships, relevant literature was collected from various medically relevant databases. The primary databases used were PubMed and Web of Science, with relevant articles from 2014 to 2024 selected. However, no restrictions were placed on the timeframe of the references to ensure a comprehensive review of the relevant literature. Our keywords were set as: curcumin, turmeric, turmeric analogs, oxidative stress, reactive oxygen species, oxidative damage, antioxidant, lipid metabolism, disordered lipid metabolism, hyperlipidemia, high lipid, dyslipidemia, hypertriglyceridemia, triglycerides, high triglycerides, hypercholesterolemia, cholesterol, high cholesterol, nonalcoholic fatty liver disease, non-alcoholic steatohepatitis, NAFLD, fatty liver, atherosclerosis, atheromatous plaque, obesity, fat accumulation, overweight, diabetes mellitus, type 2 diabetes, hyperglycemia, insulin, insulin resistance, antihyperglycemia. For the completeness and validity of the search, these keywords were adapted to meet the specific requirements of different databases. For relevant articles found in the search, an initial screening based on title and abstract was conducted to exclude articles with low or no relevance. Articles with high relevance were carefully read and summarized, with a focus on citing the results and discussion sections. Important experimental (*in vivo* and *in vitro*) and clinical studies in each disease area were summarized in tabular form.

## 3 Relationship between lipid metabolism and oxidative stress

### 3.1 Effects of oxidative stress on the pathophysiological processes of lipid metabolism

**FIGURE 2:**
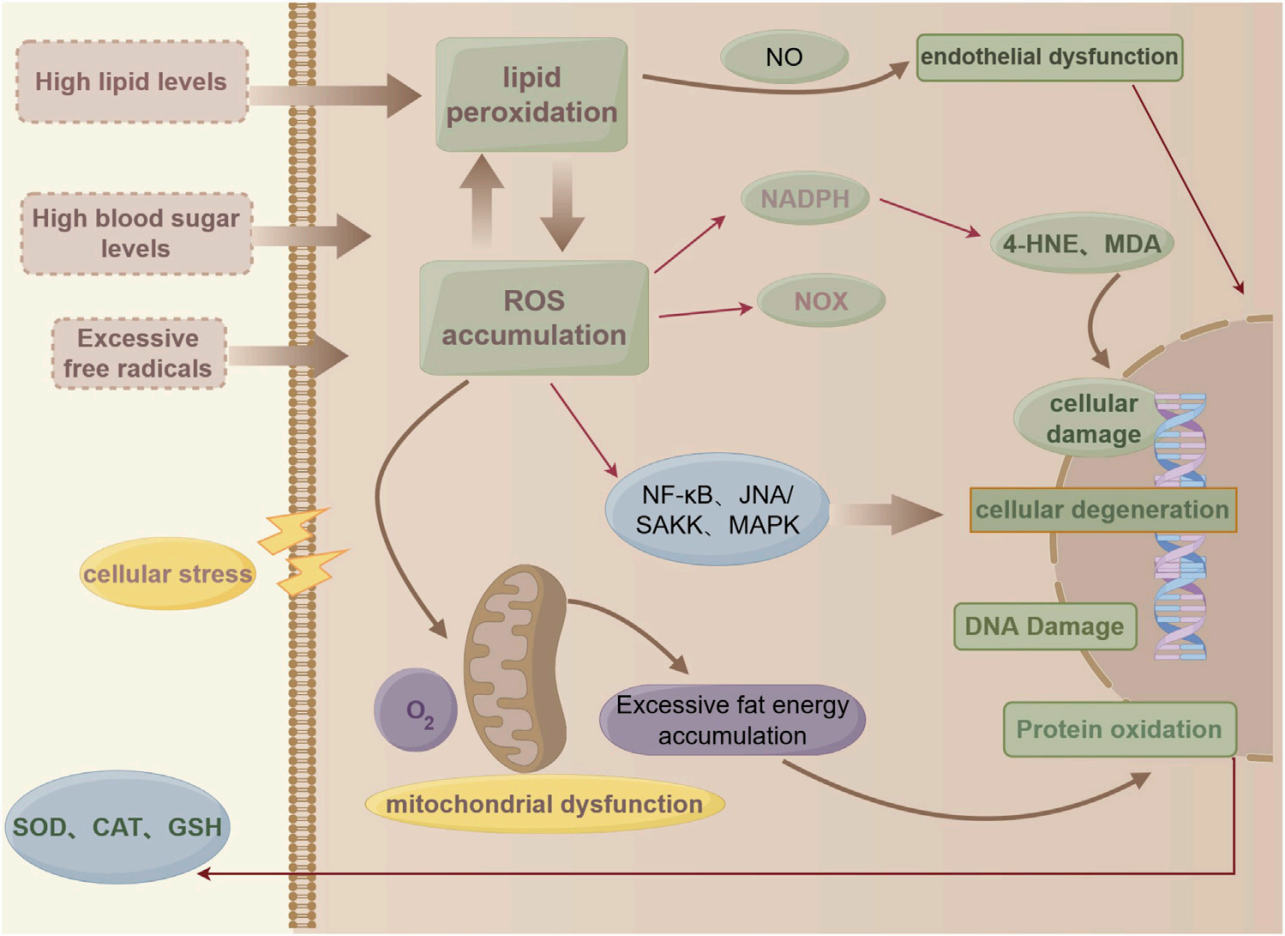
(by Figdraw: WYPUT84442): Pathological processes in lipid metabolism and oxidative stress.

The global concept of oxidative stress is “an imbalance between oxidants and antioxidants leading to molecular disruption of redox signaling and control,” and the molecular mechanisms involved in the regulation of oxidative stress include the coordination and alteration of the structure and function of proteins, lipids, carbohydrates, and nucleic acids (Sies, 2020). Under normal physiological conditions, the balance of cellular metabolism and biochemical reactions can be maintained, whereas excessive accumulation of reactive oxygen species (ROS) may induce cellular damage, particularly by damaging DNA or leading to lipid peroxidation, and may alter the expression and activity of key enzymes involved in lipid metabolism (DeVallance et al., 2019; Masenga et al., 2023). A physiological balance between lipid uptake and oxidation prevents excessive lipid accumulation; however, diseases such as diabetes and obesity alter fatty acid oxidation, resulting in lipid accumulation (Schulze et al., 2016). Lipid accumulation is a fundamental aspect of lipid metabolism disorders. Mitochondria play a crucial role in the regulation of lipid metabolism and oxidative stress, where mitochondrial ROS dysfunction disrupts metabolic signaling, promoting increased lipogenesis and reduced fatty acid β-oxidation, leading to the accumulation of triglycerides in cells (Mansouri et al., 2018). The hyperactivation of oxidative stress pathways may increase the production of lipid mediators, especially endogenous cannabinoids and arachidonates, which are lipid metabolites generated via enzymatic processes that act through specific receptors (Wójcik et al., 2021) (Figure 2).

Therefore, the relationship between oxidative stress and lipid metabolism and their effects is bidirectional. Therefore, it is feasible to intervene in oxidative stress to positively impact diseases related to lipid metabolism disorders. Therefore, we aim to identify a Chinese medicine monomer or active metabolites that can intervene in oxidative stress and simultaneously improve lipid metabolism disorders, with significant potential to address diseases associated with lipid metabolism disorders. Curcumin emerges as a promising candidate.

### 3.2 Regulatability of lipid metabolism

Lipid metabolism includes the synthesis, absorption, and storage of lipids, the catabolism, secretion, and utilization of lipids, as well as the regulation of lipid homeostasis through the coordination of these processes, referred to as the plasticity of lipid metabolism. The induction of lipid metabolism plasticity is critically influenced by changes in the stability or activity of key enzymes and core proteins. Acetyltransferase Tip60 responds to fatty acid stimulation by acetylating lipin-1, a crucial metabolic enzyme in the lipid synthesis pathway, thereby promoting triglyceride synthesis and contributing to obesity (Li et al., 2018). Under fatty acid stimulation, lipid droplet protein Cell Death Inducing DFFA Effector C (CIDEC) undergoes acetylation, resulting in reduced degradation and enhanced stability, which promotes fat storage. In turn, this process regulates metabolic homeostasis and contributes to the onset of obesity (Qian et al., 2017). Increased intracellular ROS, induced by the overaccumulation of lipids such as fatty acids and cholesterol, enhance the stability and enzymatic activity of cholesteryl ester synthase Acetyl-CoA Acetyltransferase 2 (ACAT2) through oxidation, thereby promoting cholesterol lipid production (Wang et al., 2017). Mulberry leaf water extract (MLWE) was found to play a key role in regulating lipid metabolism disorders at the genetic level by down-regulating genes involved in oxidative stress, such as acetyl coenzyme A carboxylase and fatty acid synthase while up-regulating peroxisome proliferator-activated receptor (PPARα), which encodes the PPARα. These findings suggest that MLWE has significant potential for managing lipid metabolism disorders at the genetic level (Du et al., 2022). Curcumin supplementation was found to reduce abdominal fat, plasma LDL cholesterol, and triglyceride concentrations. Additionally, it significantly decreased fatty acid synthase and sterol regulatory element-binding protein levels, while significantly increasing peroxisome proliferator-activated receptor alpha (PPARα) and carnitine palmitoyltransferase-I (CPT-I) expression in the curcumin-treated group of broilers (Xie et al., 2019). This suggests that curcumin exerts a beneficial effect on various aspects of lipid metabolism regulation. The plasticity of lipid metabolism refers to the reversibility of lipid metabolism disorders and highlights the potential for modulating metabolic diseases associated with these disorders. The elucidation of these molecular mechanisms undoubtedly provides critical targets for validating the efficacy of pharmacological interventions.

### 3.3 Lipid metabolism disorder and its pathological results

Lipid metabolism disorders are characterized by abnormalities in the synthesis, transport, metabolism, and catabolism of lipids, primarily including triglycerides, cholesterol, phospholipids, and free fatty acids (FFAs). Lipid metabolism disorders may result in abnormal synthesis or modification of lipoproteins, disrupting normal lipid transport and consequently leading to hyperlipidemia. Furthermore, reduced levels or impaired function of high-density lipoprotein (HDL) cholesterol can diminish cholesterol clearance, thereby increasing the risk of hyperlipidemia. Oxidative stress can impair the activity of lipid-metabolizing enzymes and the function of lipoproteins, exacerbating lipid metabolism disorders. Among these factors, FFAs, impaired cholesterol metabolism, and cellular ceramide lipids play a central role in mediating the onset and progression of NAFLD (Kotlyarov and Bulgakov, 2021). NAFLD has become the most prevalent liver disease globally, and its dangers extend beyond the progression to nonalcoholic steatohepatitis (NASH), cirrhosis, and HCC. Patients with NAFLD are frequently associated with dyslipidemia, dysglycemia, inflammation, and oxidative stress (Qiu Y. Y. et al., 2023). This indicates that NAFLD is not a singular disease entity but involves diverse pathological alterations that impact multiple tissues and organs as it advances (Simon et al., 2022). Integral to this process is the deterioration of the vasculature, where oxidative stress and lipid metabolism disorders lead to vascular calcification. This calcification progresses to the arteries, resulting in atherosclerosis, which forms the pathological basis of many cardiovascular diseases and increases the risk of adverse cardiovascular events (Zheng et al., 2023).

**FIGURE 3:**
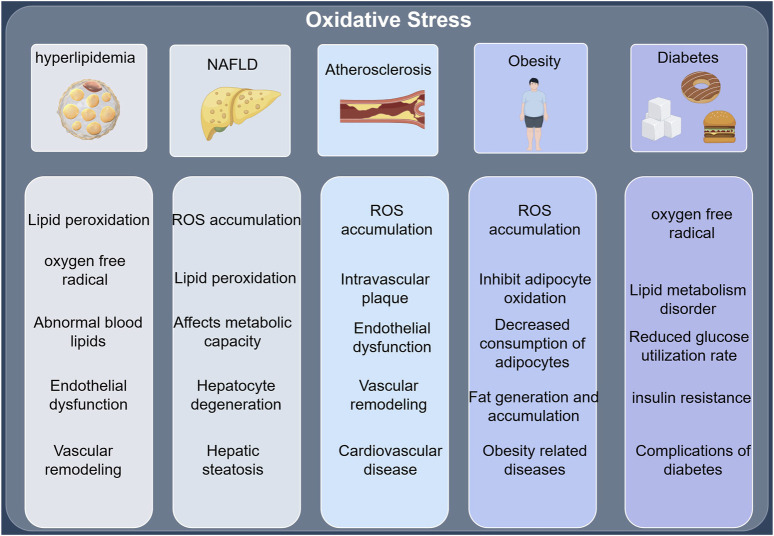
(by Figdraw: UPTPS61526): Oxidative stress and key mechanisms of hyperlipidemia, NAFLD, atherosclerosis, obesity, and diabetes.

Insulin resistance (IR) plays a critical role in the pathogenesis of dyslipidemia by disrupting the metabolism of triglycerides, high-density lipoprotein cholesterol (HDL-C), low-density lipoprotein cholesterol (LDL-C), and very low-density lipoprotein cholesterol (VLDL-C) (Bjornstad and Eckel, 2018). Insulin resistance, a central factor in the pathogenesis of type 2 diabetes mellitus, can be effectively prevented or mitigated by improving insulin sensitivity through lifestyle modifications or the pharmacological use of insulin sensitizers. Insulin resistance can further result in lipid metabolism disorders, leading to excessive fat accumulation and exacerbating obesity, with both conditions mutually reinforcing each other, forming a vicious cycle. Therapeutically, lifestyle interventions play a significant role in treating lipid metabolism disorders by positively influencing the risk factors associated with these conditions, though they have limited impact on lipid concentrations. Thus, pharmacological interventions are necessary to maximize therapeutic benefits for these patients. Adipose tissue is a crucial component of the body’s physiological homeostasis, playing a key role in glucose and lipid metabolism, which is regulated in part by the liver. The liver maintains glucose homeostasis by secreting glucose through glycogenolysis and gluconeogenesis. Consequently, liver disease can cause disturbances in hepatic glucose and lipid metabolism, contributing to the progression of type 2 diabetes (T2DM) and related metabolic disorders (Koliaki and Roden, 2013). (The key mechanisms linking oxidative stress to hyperlipidemia, NAFLD, atherosclerosis, obesity, and diabetes are shown in Figure 3).

## 4 Curcumin source, properties, and actions values

### 4.1 Sources and toxicity of curcumin

Turmeric is a perennial plant belonging to the Zingiberaceae family and the genus *Curcuma*, characterized by its fleshy, orange-colored, tuberous rhizome, and scientifically known as *Curcuma longa* L. Turmeric is widely distributed across Southeast Asia and is extensively cultivated in China, particularly in Sichuan, Yunnan, Tibet, Guangxi, and Guangdong (Akaberi et al., 2021). From the perspective of Traditional Chinese Medicine (TCM), turmeric is commonly used in TCM formulas to promote blood circulation, enhance qi flow, relieve dysmenorrhea, and alleviate pain. It is also frequently used in the treatment of oncological conditions, gynecological disorders, psychiatric illnesses, as well as metabolic diseases such as diabetes and obesity (Zhang et al., 2024). Turmeric is the most prominent natural source of curcumin, and most of its traditional applications are attributed to the presence of this key metabolites (Zeng et al., 2023). Curcumin’s pleiotropic and multi-targeted effects make it a highly attractive and beneficial polyphenolic metabolites. Curcumin has been widely used as a beverage and food additive; however, studying its toxic effects and potential adverse reactions is essential for its safe and effective application and must be given adequate attention. A 90-day chronic toxicity study of curcumin found that excessive intake enhanced glycolysis and inhibited lipid metabolism and the tricarboxylic acid (TCA) cycle in rats, leading to an imbalance in physiological homeostasis (Qiu et al., 2016). Animal studies have shown that curcumin exerts a detrimental effect on embryonic development during early pregnancy (Filardi et al., 2020). Most research on curcumin has primarily been conducted in rodent and *in vitro* studies, which have various limitations due to the differing quality of clinical studies. The lack of robust clinical data has hindered the clinical application of curcumin and similar metabolites, leaving gaps in some areas of research (Filardi et al., 2020; Zeng et al., 2022). *In vivo* and *in vitro* studies typically focus on efficacy and other positive outcomes; however, the study of adverse drug reactions and toxicity should not be overlooked. Ignoring these aspects is dangerous and must be taken more seriously by researchers.

### 4.2 Basic properties and pharmacokinetics of curcumin

**FIGURE 4:**
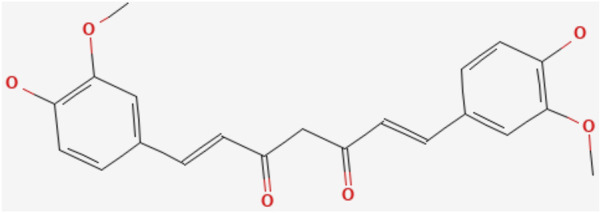
(by PubMed): Curcumin chemical formula.

Curcumin, a metabolite discovered over a century ago, is structurally characterized by a seven-carbon chain in which α,β-unsaturated β-diketones are partially linked to two phenyl rings with o-methoxy groups. It exists in both diketone and enolized reciprocal isomeric forms, with the diketone form predominating in solid-phase, acidic, and neutral conditions, while the enolone form dominates in alkaline environments (Noureddin et al., 2019; Priyadarsini, 2013). (The chemical formula of curcumin is shown in Figure 4). The metabolic pathways of curcumin metabolites *in vivo* encompass phase I reduction metabolism, phase II conjugation metabolism, autoxidation, and intracellular catalytic oxidative metabolism (Zhou et al., 2024). Key chemical reactions, including hydrogen donation, reversible and irreversible nucleophilic addition (Michael reactions), hydrolysis, degradation, and enzymatic oxidation of curcumin, are linked to its unique biological activities. Curcumin’s oxidative properties enable it to scavenge ROS *in vivo*, conferring antioxidant activity in normal cells (Priyadarsini, 2014).

The solubility and stability of curcumin depend on the type of solvent and pH level. Curcumin is highly insoluble in water but soluble in organic solvents. It is relatively stable under acidic to neutral conditions but becomes very unstable and prone to decomposition under alkaline conditions (Nelson et al., 2017). It has been shown that curcumin is poorly absorbed in the gastrointestinal tract after oral administration, with only a small amount entering the peripheral blood circulation through the portal vein (Yang et al., 2024). In an early study investigating the uptake, distribution, and excretion of curcumin in SD rats, it was observed that following oral administration of curcumin (1 g/kg), approximately 75% of the curcumin was excreted in the feces, with negligible amounts in the urine. Additionally, 90% of the administered curcumin was metabolized in hepatocyte suspensions within 30 min (Wahlström and Blennow, 1978). In another clinical trial, oral administration of curcumin (4 g/day for 30 days) to 19 subjects revealed that trace amounts of curcumin were detected in the serum of only 2 subjects, with a mean blood concentration of just (3.8 ± 1.3 ng/mL) (Carroll et al., 2011). This phenomenon is attributed to the first-pass effect of the enterohepatic circulation following oral administration of curcumin. Additionally, when curcumin-like formulations are administered orally in humans, few adverse events are observed, and they are generally well tolerated, suggesting that curcumin or curcumin-like metabolites may be safe as low-dose phytopharmaceutical supplements (Nelson et al., 2017). However, plasma concentrations of curcumin are significantly higher following injection. Intraperitoneal injection of curcumin (single dose of 100 mg/kg) was found to remain *in vivo* in tissues and plasma for 2–8 h (Perkins et al., 2002), Pan et al. injected curcumin (100 mg/kg) into the peritoneum of mice, and high plasma levels of curcumin (2.25 μg/mL) were achieved within 15 min (Pan et al., 1999). Additionally, with the advancement of drug delivery systems, various curcumin nano-formulations, including liposomes, polymers, nanoparticles, conjugates, cyclodextrins, and nanogels, have been employed to enhance the bioavailability of curcumin to varying extents (Jabczyk et al., 2021).

### 4.3 Antioxidant effects of curcumin

Curcumin ameliorates lipid metabolism disorders by exerting antioxidant activity and reducing the accumulation of ROS. This includes reducing fat deposition, enhancing fatty acid uptake, and alleviating insulin resistance. Curcumin can scavenge a variety of reactive oxygen species, such as hydrogen peroxide, nitric oxide (NO), and superoxide radicals, and exerts its antioxidant effects by preventing lipid peroxidation (Ak and Gülçin, 2008). Additionally, curcumin enhances the activity of endogenous antioxidant enzymes, such as superoxide dismutase (SOD) and CAT, thereby boosting the antioxidant capacity of cells. Intracellular ROS are primarily regulated by the endogenous antioxidant defense system. Curcumin reduces the expression of pro-inflammatory factors by inhibiting the activation of nuclear factor κB (NF-κB), thereby mitigating the inflammatory response and oxidative stress. Furthermore, curcumin inhibits ROS production, enhancing the antioxidant capacity of cells (Das and Vinayak, 2012).

Curcumin is intricately involved in oxidative stress pathways, as demonstrated by its ability to inhibit Kelch-like ECH-associated protein 1(Keap1), modulate the NF-E2-related factor2(Nrf2) signaling pathway, and promote the transcription of the Antioxidant Response Element (ARE). This upregulates the expression of various antioxidant enzymes (e.g., NQO1, HO-1, and GST), which clear free radicals in the body, thereby enhancing cellular antioxidant activity and mitigating oxidative stress. These properties make curcumin a promising candidate for the treatment of several oxidative stress-related diseases (Ashrafizadeh et al., 2020; Niture et al., 2010; Rahban et al., 2020). Therefore, supplementation with antioxidants like curcumin to scavenge free radicals and reduce oxidative stress, thereby protecting cells from damage, holds significant importance and potential for disease treatment. Together, these mechanisms allow curcumin to exhibit significant pharmacological effects, including antioxidant, anti-inflammatory, and antitumor activities, while also providing a theoretical basis for its use in clinical applications.

## 5 The effect of curcumin on lipid metabolism disorders and related diseases by improving oxidative stress

### 5.1 Hyperlipidemia

#### 5.1.1 Dysregulation of lipid metabolism and hyperlipidemia

Hyperlipidemia is a metabolic disorder characterized by excessively high lipid levels in the blood. These elevated lipid levels provide abundant substrates for free radicals, which attack unsaturated fatty acids, leading to lipid peroxidation. The intensification of lipid peroxidation generates large amounts of ROS, thereby activating the oxidative stress pathway (Gaschler and Stockwell, 2017; Yao et al., 2020). Activation of oxidative stress, in turn, intensifies lipid peroxidation, which not only worsens hyperlipidemia but also contributes to the progression of other diseases related to lipid metabolism disorders. Furthermore, hyperlipidemia leads to endothelial dysfunction, increases vascular permeability, and triggers an inflammatory response, further exacerbating oxidative stress (Xu et al., 2021). Hyperlipidemia is a major contributing factor in the occurrence, development, and prognosis of cardiovascular diseases. Therefore, supplementation with antioxidants like curcumin to scavenge free radicals and reduce oxidative stress, thereby protecting cells from damage, holds significant promise for the treatment of cardiovascular diseases. However, the molecular mechanisms and specific targets of action require further study and elucidation.

#### 5.1.2 Reduce lipid levels

The potential mechanisms by which curcumin ameliorates dyslipidemia include the reduction of protein lipase activity, inhibition of fatty acid synthase (FAS) activity, enhancement of fatty acid β-oxidation, and possible inhibition of cholesterol synthesis through modulation of hepatic enzyme activity (Babu and Srinivasan, 1997; Shehzad et al., 2011). Curcumin may ameliorate hyperlipidemia and insulin resistance in high-fat diet-fed hamsters by lowering triglycerides, FFAs, total cholesterol, and homeostasis model assessment of insulin resistance (HOMA-IR). Curcumin was found to reduce serum total cholesterol (TC) and triglyceride (TG) levels and improve oxidative stress by decreasing malondialdehyde (MDA) and increasing SOD, glutathione (GSH), and CAT levels in the kidneys of rats with passive Heymann nephritis (PHN). These effects may be mediated through modulation of the Nrf2/HO-1 signaling pathway (Di Tu et al., 2020). Curcumin pretreatment in hyperlipidemic rats reduced hepatic lipid damage by 40% and renal lipid damage by 56%, suggesting that curcumin restored immune homeostasis and redox balance in peripheral organs disrupted by hyperlipidemia (Manzoni et al., 2020).

#### 5.1.3 Reduce the harm of hyperlipidemia

Studies have shown that absolute and relative abdominal fat weight, plasma LDL cholesterol concentration, and plasma and hepatic triglyceride levels were significantly reduced in broilers fed a diet supplemented with 2,000 mg/kg of curcumin (Xie et al., 2019). ATP-citrate lyase (ACLY) functions upstream of acetyl coenzyme A carboxylase, converting citrate derived from the tricarboxylic acid cycle into cytoplasmic acetyl coenzyme A and oxaloacetate, thereby contributing to fat accumulation and the progression of fatty liver (Feng et al., 2020). ACLY inhibition was found to reduce hepatic malonyl coenzyme A, oxaloacetate, steatosis, and ballooning, as well as blood glucose, triglycerides, and cholesterol levels (Morrow et al., 2022). The gene expression levels of acetyl coenzyme A carboxylase (ACC) and ACLY were significantly reduced in broilers fed a diet supplemented with 2,000 mg/kg of curcumin. These findings suggest that curcumin plays an important role in reducing abdominal fat deposition by lowering liver and plasma lipid levels and modulating the expression of genes involved in lipogenesis and lipolysis, such as ACC and ACLY (Xie et al., 2019).

### 5.2 NAFLD

#### 5.2.1 Oxidative stress and the onset and progression of NAFLD

The pathogenesis of NAFLD has not been fully elucidated, but at its core, FFAs derived from triglyceride catabolism in adipose tissue are transported to the liver through the bloodstream. This transport results in damage from fatty acid β-oxidation and mitochondrial dysfunction. Combined with an imbalance between hepatic fat production and degradation, this leads to reduced metabolic capacity in the liver, ultimately resulting in hepatic steatosis (Chen et al., 2019; Guo et al., 2022). This, combined with obesity, type 2 diabetes, dyslipidemia, and other adverse effects on the liver, is referred to as the “first strike.” The “second strike” involves oxidative stress and pro-inflammatory cytokines, leading to systemic hepatocellular damage and liver inflammation (Musso et al., 2013). ROS, as key signaling factors in the development of NAFLD, mediate multiple responses to lipid peroxidation, leading to elevated levels of peroxides such as 4-hydroxy-2-nonenal (4-HNE) and MDA. These peroxides activate Kupffer cells (KCs) and hepatic stellate cells (HSCs), and the interactions between these cytokines form a vicious cycle in which increased ROS production and accumulation result in sustained hepatocyte degeneration and injury (Gong et al., 2024; Guariglia et al., 2023).

Oxidative stress is linked to sustained hepatocyte damage, characterized by an increase in oxygen free radicals, ROS accumulation, and a decrease in antioxidant defenses. Prolonged exposure to this chronic stress gradually leads to structural changes in the cells and the oxidation of substrates such as lipids, proteins, and DNA, ultimately impairing cellular function (Cichoż-Lach and Michalak, 2014; Rezzani et al., 2019). Curcumin and its related metabolites inhibit the formation of oxygen radicals and exhibit ROS-scavenging effects, thereby reducing DNA damage (Amalraj et al., 2017).

#### 5.2.2 Positive effects on various stages of NAFLD

Elevated FFAs resulting from excessive dietary fat intake are a key risk factor for fatty liver. As excess FFAs are taken up by the liver and gradually accumulate, FFA uptake factors such as CD36 are activated, leading to an imbalance in hepatic ROS and histone deacetylase 2 (HDAC2). This imbalance may promote the onset and progression of NASH (Zhong et al., 2017). Among the various pathogenic mechanisms of NAFLD, oxidative stress is regarded as a major contributing factor. Curcumin aqueous extract (CLW) was found to upregulate CPT-1 and PPAR-α, suggesting that CLW contributes to increased β-oxidation, thereby reducing lipid accumulation in hepatocytes (Mun et al., 2019). In an animal study, curcumin was found to reverse the expression of p67phox and p-ERK1/2 in Nicotinamide Adenine Dinucleotide Phosphate (NADPH) oxidase enzymes, alleviating liver injury in NASH-HCC mice by reducing oxidative stress (Afrin et al., 2017). Additionally, curcumin attenuated hepatic steatosis induced by a high fructose diet (HFHFr) in NAFLD mice, as evidenced by reversing the expression of CYP3A and CYP7A (exogenous and endogenous metabolizing enzymes) and normalizing serum biochemical parameters (TC, TG, and NEFA) in the fatty liver state. These effects contributed to the restoration of hepatic metabolic capacity (Yan et al., 2018).

8-hydroxy-2′-deoxyguanosine (8-OHdG) is a potential marker of oxidative DNA damage, and the oxidative stress state in NAFLD leads to the overproduction of advanced glycation end products (AGEs). Excessive AGEs bind to hepatocytes and damage them, further exacerbating oxidative stress (Mashayekhi-Sardoo et al., 2021; Zeng et al., 2023). A randomized controlled trial demonstrated that supplementation with phospholipid curcumin capsules (8 mg/day) reduced levels of 8-OHdG and carboxymethyl lysine (CML), one of the end products of AGEs, in NAFLD patients (Mirhafez et al., 2019). Additionally, a combined clinical and epigenetic trial involving 54 NAFLD patients found that phytosome curcumin supplementation (250 mg/day for 8 weeks) reduced promoter methylation of MLH1 and MSH2(Two important mismatch repair proteins), thereby lowering the risk of base pair mismatches in the DNA of NAFLD patients (Hariri et al., 2020). In the context of hepatitis and oxidative stress, the interaction and dysfunction of hepatocytes, macrophages, and hepatic stellate cells (HSCs) are closely associated with the development of NAFLD (Bae et al., 2018). These findings suggest that curcumin exerts beneficial effects on various aspects of oxidative stress in NAFLD.

### 5.3 Atherosclerosis

#### 5.3.1 Pathological basis

Atherosclerosis is the pathological basis for many cardiovascular diseases and acute cardiovascular events, including most cases of coronary artery disease, myocardial infarction, stroke, and peripheral arterial disease (Libby et al., 2019). Atherosclerosis primarily arises from metabolic dysregulation and the accumulation of excess lipids in macrophages during lipid transport, leading to the formation of foam cells. These foam cells can form and exacerbate unstable plaques when they are significantly retained in the vascular endothelium (Luo et al., 2017; Wang et al., 2023). One of the key events in the development of atherosclerosis is endothelial dysfunction, which makes certain regions prone to atheromatous plaques. These plaques are strongly associated with hemodynamic alterations and oxidative stress caused by the accumulation of ROS (Gimbrone and García-Cardeña, 2016; Kattoor et al., 2017). The major ROS-generating systems in the vascular wall include NADPH oxidase, xanthine oxidase, the mitochondrial electron transport chain, and uncoupled endothelial nitric oxide (NO) synthase (Förstermann et al., 2017).

#### 5.3.2 Endothelial dysfunction

It has been shown that feeding mice a curcumin chow mix (0.1% curcumin + HFD) not only controls body weight gain but also enhances HO-1 enzyme activity in aortic tissues and maintains Sirt1 expression, contributing to the inhibition of oxidative stress in the vasculature and throughout the body (Takano et al., 2018). Another study found that in rats fed a high-sucrose, high-fat diet, curcumin supplementation inhibited inflammation and oxidative damage in vascular endothelial cells by enhancing NO production and increasing catalase (CAT) and GSH activity (Tsai et al., 2018). Additionally, curcumin may combat atherosclerosis by exerting anti-inflammatory and antioxidant effects, as well as inhibiting the proliferation and migration of vascular smooth muscle cells (VSMCs) (Singh et al., 2021). Plasma lipid levels and lipid peroxidation status are crucial factors contributing to atherosclerosis, and curcumin plays a protective role against atherosclerosis by counteracting lipid peroxidation (Cox et al., 2022).

Early experimental studies demonstrated that supplementation with a hydroalcoholic extract of curcumin reduced aortic lipid streak formation in hypercholesterolemic diet-fed (HDF) rabbits and lowered systemic oxidative stress (Quiles et al., 2002). It is well known that hypertension is one of the major causes of atherosclerosis, contributing to endothelial damage, increasing vascular resistance, and promoting fat deposition. Curcumin has been found to attenuate hypertension-induced vascular oxidative stress and improve endothelial dysfunction, offering protective effects against both hypertension and atherosclerosis (Boonla et al., 2014). Peroxisome proliferator-activated receptor γ (PPAR-γ) is a key regulator of glucose homeostasis, lipid metabolism, and endothelial cell proliferation (Meng et al., 2014; Qiu Y. Y. et al., 2023). Curcumin has been shown to inhibit IL-6 and TNF-α expression by increasing PPAR-γ activity and attenuating oxidative stress, reducing NO production, and ultimately inhibiting the proliferation of vascular smooth muscle cells (VSMCs), while also reducing intracellular ROS accumulation (Li et al., 2017).

### 5.4 Obesity

#### 5.4.1 Obesity and oxidative stress

Obesity is defined as a body mass index (BMI) greater than 30, and those with a BMI between 25 and 29.9 are considered overweight; however, defining obesity based solely on BMI is inadequate and may hinder obesity prevention and intervention efforts. Oxidative stress plays a critical role in the pathophysiology of obesity by increasing the inflammatory response in adipose tissue and stimulating the differentiation of preadipocytes into mature adipocytes, thereby inhibiting fatty acid oxidation (FAO) and ultimately promoting adipogenesis (Kasprzak-Drozd et al., 2022). Obesity not only affects lipid levels but also alters the composition and structure of various adipose tissues, such as brown adipose tissue (BAT) and perivascular adipose tissue (PVAT). This adipose tissue dysfunction may induce systemic oxidative stress and impact the progression and prognosis of diseases such as T2DM and metabolic syndrome (Cox et al., 2022; Marseglia et al., 2014). In addition, excess nutrient supply can inhibit the Krebs cycle and the mitochondrial respiratory chain, resulting in mitochondrial dysfunction and increased ROS accumulation, and conversely, both can reduce adipocyte O2 consumption, ultimately promoting energy storage in adipose tissue (Bondia-Pons et al., 2012; de Mello et al., 2018; Wang et al., 2010).

Standardized obesity management is a long-term, comprehensive, and individualized approach, with the main therapeutic strategies including lifestyle interventions, anti-obesity medications, and bariatric surgery. However, the long-term efficacy and safety of anti-obesity medications are currently under scrutiny (Müller et al., 2022; Perdomo et al., 2023). Curcumin shows significant potential in obesity treatment, as evidenced by its ability to promote white fat conversion and its positive effects on glycemic status and insulin sensitivity. It can also reduce adipose tissue inflammation associated with obesity by scavenging ROS while also blocking NF-κB (nuclear factor-κB) activation and reducing target gene expression (Cox et al., 2022; Pérez-Torres et al., 2021).

#### 5.4.2 Clinical trials

A randomized, placebo-controlled trial involving 60 obese or overweight adolescent girls found that a daily intake of 500 mg of curcumin for 10 weeks enhanced total antioxidant capacity (TAC) and decreased MDA levels (Saraf-Bank et al., 2019). However, the trial was limited because it only addressed correlative indicators and did not examine obesity outcome indicators. In addition, Sahebkar et al. investigated the effect of curcumin supplementation on serum pro-oxidant-antioxidant balance (PAB) and antioxidant low-density lipoprotein (ox-LDL) antibody titers and demonstrated that oral curcumin supplementation (1 g/day for 30 days) effectively reduced oxidative stress levels (Sahebkar et al., 2013). A meta-analysis demonstrated that curcumin intake was associated with significant decreases in BMI, body weight, waist circumference, and leptin, along with increases in lipocalin levels (Akbari et al., 2019). A randomized controlled trial demonstrated that curcumin supplementation positively affected adipokines, including lipocalin and leptin, in obese individuals and led to reductions in body weight and adiposity in the curcumin group (1 g/day) (Panahi et al., 2016). In addition, mice were fed a very high-fat diet (VHFD) supplemented with 0.7% curcumin for 14 weeks and exhibited decreased body weight and average fat content compared to the control group (Koboziev et al., 2020).

In conclusion, curcumin improves obesity-related metrics, including reductions in body weight, BMI, and waist circumference, and enhances adiposity-related factors. Reducing adiposity is a fundamental intervention to improve lipid metabolism, which is crucial for addressing lipid metabolism-related disorders such as hyperlipidemia, diabetes mellitus, and non-alcoholic fatty liver disease.

### 5.5 Diabetes

#### 5.5.1 Diabetes and disorders of lipid metabolism

Diabetes decreases glucose utilization and increases the levels of oxygen-derived free radicals, resulting in an imbalance between antioxidant capacity and free radical and/or reactive oxygen species levels, ultimately leading to oxidative stress in the body (Zhang Y. et al., 2016). In addition, abnormal lipid metabolism plays a role in the progression of diabetes as a result of insulin resistance and abnormal metabolic changes that disrupt key enzymes and lipid metabolic pathways (Wu and Parhofer, 2014). Increased oxidative stress significantly affects the progression and prognosis of diabetic complications, such as macrovascular and microvascular complications, which are associated with abnormal lipid metabolism and recurrent transient hyperglycemic episodes (Lima et al., 2020). Regular intake of curcumin has been found to improve glucose and lipid metabolism, enhance intracellular antioxidant responses, and improve insulin signaling, providing benefits for diabetes-related metabolic diseases (Kim and Clifton, 2018).

#### 5.5.2 Basic research and clinical trials

In a human trial lasting 8 weeks, curcumin analogs were shown to reduce serum malondialdehyde (MDA) levels and significantly increase serum TAC and SOD activity in patients with T2DM (Panahi et al., 2017). Another animal study demonstrated that curcumin alleviated oxidative stress in a rat model of type 1 diabetes (STZ-induced), as evidenced by not only lowering blood glucose levels, but also decreasing plasma MDA, GSH-Px, and CAT activity while increasing SOD and insulin levels (Xie et al., 2018). The value of curcumin in preventing and reducing oxidative stress and mitochondrial dysfunction was demonstrated by findings showing that curcumin treatment improved mitochondrial TBARS levels in the liver and kidney in experiments with animal models of hyperglycemia, and may enhance oxygen consumption rate and ATPase activity through scavenging free radicals and preventing protein and lipid peroxidation (Soto-Urquieta et al., 2014). High doses of curcumin reduced TC, TG, LDL-C, and HDL-C levels in diabetic rats by lowering fasting blood glucose (FGB) concentrations improving oral glucose tolerance, and preventing its deterioration in diabetic rats (Xia et al., 2020). In an animal study, curcumin treatment reduced fasting blood glucose levels and increased insulin levels in diabetic rats, and significantly reduced cholesterol and triglyceride levels (Alsulaim et al., 2023). Curcumin attenuated high glucose/palmitate (HP)-induced oxidative stress in pancreatic islet cells and reduced apoptotic damage in these cells by modulating the NADPH pathway (Li et al., 2019).

#### 5.5.3 Beneficial effects on diabetes complications

The developmental characteristics of diabetes mellitus cause its late-stage complications to significantly impact the survival and quality of life of diabetic patients. Therefore, addressing the complications of diabetes is an important part of comprehensive diabetes management. Dehydrozingerone (DHZ), a curcumin analog, has been shown to reduce the incidence of high-fat diet (HFD)-induced diabetic nephropathy (DN) in an obese mouse model by down-regulating lipid accumulation and ROS production in the kidney (Lee et al., 2021). Diabetic gastroparesis (DG) is mainly characterized by delayed gastric emptying (GE) due to impaired non-adrenergic, non-cholinergic relaxation of the stomach, which may be associated with reduced or absent Nrf2 levels. Curcumin has been shown to restore gastric emptying (GE) by activating Nrf2 to suppress oxidative stress markers and pro-inflammatory cytokines in the gastric tissues of obesity-induced diabetic mice (Bharucha et al., 2019; Sampath et al., 2021). Supplementation with curcumin also inhibited myocardial lipid peroxidation and transforming growth factor β1 (TGF-β1) production in diabetic cardiomyopathy rats, which attenuated oxidative stress and myocardial fibrosis (Gbr et al., 2021). Nanocurcumin has the potential to attenuate cardiac inflammation and programmed cardiomyocyte death by inhibiting oxidative stress and advanced glycosylation end products (AGEPs) in the cardiac tissues of diabetic rats (Abdel-Mageid et al., 2018; Table 1).

**TABLE 1:** Experimental study of curcumin intervention in oxidative stress to ameliorate diseases associated with lipid metabolism disorders.

References	Treatment	Model	Intervention dose	Control groups	Periods	Results	Machine
Manzoni et al. (2020)	Curcumin	Wistar rat	Pre-treatment: 50 mg/kg Hyperlipidemia group: 50 mg/kg	cold, sterile 0.9% NaCl solution	30 days	Decreased SOD and CAT levels in both curcumin-pretreated and hyperlipidemic groups	Curcumin enhances the antioxidative stress defense mechanism
Wu et al. (2023)	Curcumin	Male Cobb Broiler	Basic dietary feeding of 50, 100 and 150 mg/kg of curcumin	Basal diet without curcumin supplementation	21 days	Increased TAOC activity and decreased MDA content in 150 mg/kg curcumin group	Curcumin exhibits antioxidant activity, free radical scavenging capacity, and Nrf2-mediated stress mitigation
Lee et al., 2021	Dehydrozingerone (DHZ)-Curcumin analog	C57BL/6 mice	DHZ (100 mg/kg)	Group I: Regular diet Group II: HFD (60% kcal from fat	12 Weeks	Reduced PA-induced NOX4 expression, enhanced antioxidant signals (Nrf2/HO-1), and modulated apoptosis markers (BAX/Bcl2)	Curcumin modulates oxidative stress and lipid metabolism
Wu et al. (2021)	Curcumin	Ducks	400 mg/kg curcumin	Group I: 4 mg/kg ATO Group II: 8 mg/kg ATO	28 days	Decreased MDA, upregulated Nrf2 expression, and reduced Trx and HO-1 levels in curcumin intervention groups	Curcumin alleviates excessive autophagy, apoptosis, and lipid metabolism disorders by modulating oxidative stress
Silva-Gaona et al. (2022)	Curcumin	C57BL6J male mice	30% w/v fructose in drinking water +0.75% w/w curcumin in food	Group I: Standard diet Group II: 0.75% w/w curcumin	15 weeks	Curcumin mitigated changes in cellular pathways related to oxidative phosphorylation (NDUFB8, NDUFB3, ATP5L) and lipid metabolism (THRSP, DGAT1, ECI1, ACOT13)	Curcumin regulates protein expression and metabolic pathways
Afrin et al., 2017	Curcumin	C57BL/6J male mice	Curcumin 100 mg/kg/day (NASH + Curcumin)	Group I: Normal diet NASH group (STZ-injected mice)	4 weeks	Reduced C/EBPβ and CYP2E1 levels, enhanced Nrf2 expression, and reversed p67phox and p-ERK1/2 expression	Curcumin attenuates oxidative stress in NASH-HCC liver
Hariri et al. (2020)	Phytosome curcumin (Meriva, 250 mg phosphorylated curcumin = 50 mg curcumin)	54 patients with NAFLD	Meriva (250 mg phospholipidated curcumin equivalent to 50 mg curcumin)	placebo capsules	8 weeks	Reduced promoter methylation of MLH1 and MSH2 in NAFLD patients	Curcumin reduces the risk of base pair mismatches in the DNA of NAFLD patients
Sahebkar et al. (2013)	curcuminoids	Thirty obese individuals	Curcuminoids 1g/day	placebo	30 days	Curcuminoids supplementation reduces PAB levels	Curcuminoids reduces the burden of oxidative stress
Saraf-Bank et al., 2019	Curcumin	60 overweight or obese adolescent girls	Curcumin 500 mg/day	placebo capsules	10 weeks	Curcumin resulted in increased TCA levels and decreased MDA levels	Curcumin has beneficial effects on markers of oxidative stress
Takano et al., 2018	Curcumin	Male C57BL/6J mice	0.1% curcumin added to feed	standard feed); HFD group (fed with HFD)	10 weeks	Inhibited weight gain, increased HO-1 expression, and maintained Sirt1 expression	Curcumin inhibits systemic and vascular oxidative stress
Alsulaim et al. (2023)	Curcumin	T2DM rats	Oral curcumin: 50 mg/kg/day	Normal control: saline Negative control: STZ-induced diabetic rats Positive control: treated with glibenclamide	8 weeks	Reduced TC and TG levels, and enhanced antioxidant enzymes (CAT, GST, SOD) in curcumin-treated groups	Curcumin enhances antioxidant defenses and mitigates lipid peroxidation
Xia et al. (2020)	Curcumin	male SD rats	Curcumin100 mg/kg/day or 300 mg/kg/day	HFD + STZ (20 mg/kg body weight)	8 weeks	Upregulated SOD, CAT, and GSH levels, while MDA levels were reduced in curcumin intervention groups	Curcumin may prevent or treat T2DM by enhancing antioxidant activity

## 6 Discussion

### 6.1 The potential of curcumin

This paper provides a comprehensive analysis of the research progress on curcumin in attenuating oxidative stress and regulating lipid metabolism disorders and related diseases. Curcumin, a natural polyphenolic metabolites, has been shown in a systematic review of the literature to significantly reduce oxidative stress and exhibit antioxidant effects through multiple biological pathways, including inhibition of reactive oxygen species generation, upregulation of antioxidant enzyme expression, and regulation of lipid metabolism-related gene expression. These findings not only enhance our understanding of curcumin’s biological activities but also offer a scientific basis for the development of novel therapeutic strategies against hyperlipidemia, NAFLD, atherosclerosis, diabetes, and obesity. Curcumin exhibits significant therapeutic effects, regulating blood lipids and blood glucose levels, improving insulin resistance, alleviating vascular endothelial dysfunction, inhibiting fatty acid synthesis, promoting fat conversion, protecting the liver from oxidative stress damage, and promoting liver repair.

### 6.2 Limitations of the action of curcumin

Curcumin is a widely studied bioactive metabolite with medicinal properties, including anti-inflammatory, antioxidant, and antitumor effects, which have attracted significant attention in scientific research. However, its clinical application is hindered by low bioavailability, rapid degradation, and poor water solubility, all of which contribute to its limited oral bioavailability and reduced clinical efficacy (Dei Cas and Ghidoni, 2019; Moetlediwa et al., 2023). To address these limitations, researchers have developed various strategies to enhance curcumin’s effectiveness. These include the development of curcumin delivery systems, the design and synthesis of curcumin analogs or derivatives, and the co-administration of other substances alongside curcumin, among others (Karthikeyan et al., 2020; Muthumani and Miltonprabu, 2015; Panahi et al., 2015). All these strategies aim to maximize the properties and bioavailability of curcumin. Although research on curcumin delivery systems is relatively advanced and highly sophisticated, most of these systems remain at the conceptual or experimental stage and require further clinical observational studies to validate their efficacy. Additionally, techniques and methods for improving curcumin’s bioavailability, as well as addressing its adverse effects and cost-related challenges, still warrant further exploration (Table 2).

**TABLE 2:** Experimental studies to improve the bioavailability of curcumin and its key metabolites.

References	Treatment	Model	Intervention dose	Control groups	Periods	Results	Machine
Elbaset et al. (2022)	Curcumin nanoemulsion (CUR)	Wistar rats	CUR nanoemulsion 5 and 10 mg/kg	conventional powdered CUR 50 mg/kg	2 weeks	C UR nanoemulsion counteracts oxidative and nitrosative stress, DNA oxidative damage, and disturbed cellular energy status in the liver and heart	Higher doses of CUR nanoemulsion provide superior amelioration of insulin resistance and hyperlipidemia
Boshagh et al. (2023)	curcumin-piperine supplementation	66 patients with stroke	500 mg curcumin +5 mg piperine	placebo tablets	12 weeks	Intervention group exhibited higher TAC and lower TC and TG levels compared to the control group	Curcumin-piperine supplementation had beneficial effects on CIMT, TC, TG, TACin patients with ischemic stroke in the rehabilitation stage
Panahi et al. (2015)	curcuminoid-piperine combination	117 subjects with MetS	Curcumin 1 g/day + piperine 10 mg/day	matched placebo	8 weeks	Improved SOD activity and reduced MDA levels in the curcuminoid-piperine group	Curcuminoid-piperine combination significantly improves oxidative and inflammatory status in MetS patients
Feng et al. (2018)	Combination of curcumin and berberine	Male SD rats	Berberine 50 mg/kg + Curcumin 50 mg/kg	Model control (0.5% sodium carboxymethylcellulose); Berberine group (100 mg/kg bw)	8 weeks	The combination reduced lipid levels, MDA, SREBP-1c, and NF-κB more effectively	Curcumin and berberine combination improves oxidative stress, liver inflammation, and lipid metabolism
Bulboacă et al. (2019)	Liposomal Curcumin (ICC)	Wistar-Bratislava rats	ICC pre-treatment 2 mg/100 g bw	Non-diabetic group; STZ-induced DM (60 mg/100 g bw)	28 days	ICC pre-treatment significantly reduced nitric oxide, MDA, total oxidative stress, and catalase levels	ICC improved antioxidant capacity and shows potential as an adjuvant therapy for diabetes
Muthumani and Miltonprabu (2015)	Tetrahydrocurcumin (THC)	Rats	THC was pretreated at 20,40 and 80 mg/kg	Control rats; Arsenic treated rats administered with NaAsO2 (5 mg kg−1 bw)	28 days	THC restores arsenic-induced changes in serum lipid levels, increases antioxidant enzyme activity	THC reduced oxidative stress, dyslipidemia, mitochondrial damage, and arsenic-induced liver injury in rats
Yu et al. (2018)	Dihydrocurcumin (DHC)	L02 and HepG2 cells	DHC pre-treatment (5, 10, 20 μM)	Human normal liver cell line L02 and human hepatocellular carcinoma cell line HepG2	24 h	DHC decreased intracellular ROS and TG levels, inhibited SREBP-1C and PNPLA3 expression, and upregulated PPARα expression	DHC modulates lipid metabolism, oxidative stress, and insulin resistance to alleviate hepatic steatosis

### 6.3 The safety and efficacy of curcumin

Curcumin is a safe antioxidant with diverse pharmacological activities, and its efficacy and safety have been demonstrated in many animal studies and clinical observations. However, its safety profile and potential drug-drug interactions should still be considered in practical applications, and curcumin should be used under the supervision of a physician in the presence of liver disease and people undergoing treatment. In addition, there is limited data from clinical trials to fully establish the efficacy, adverse effects, and benefits of curcumin in the treatment of clinical diseases. Moreover, at high concentrations, curcumin may also exhibit pro-oxidant effects, which may lead to cellular damage. In the future, more human data are needed to better evaluate the efficacy of curcumin in different diseases, as well as more scientific studies to determine the optimal conditions for curcumin’s efficacy. In addition to animal studies and clinical research, there are many systematic evaluations and meta-analyses confirming curcumin’s efficacy and safety in combating oxidative stress (Qiu L. et al., 2023). However, such studies also have research limitations, including the inclusion of a single population, a limited number of clinical studies, and the uncertain bioavailability of curcumin, which hinders the optimization of its clinical application.

### 6.4 Discrepancies between research and clinical application

A review of the current literature reveals that animal models for lipid metabolism-related diseases are primarily induced through specialized diets, such as high-fat diet (HFD), high-fat and high-sugar diet, or by using genetically modified animals with relevant gene knockouts. The existing literature primarily focuses on liver injury or fatty liver resulting from excessive lipid droplet accumulation, while fewer studies examine the underlying causes of abnormal lipid metabolism that contribute to similar liver injuries. This indicates the need for further research to develop animal models for diseases associated with clinical lipid metabolism disorders. Furthermore, much of the research on curcumin has concentrated on animal studies, and the results have yet to be consistently validated in clinical trials. The discrepancies in clinical trial outcomes may be attributed to factors such as sample size, study design, and population heterogeneity, which undermine the feasibility of using curcumin for the treatment of human diseases. Although curcumin has demonstrated significant biological benefits in preclinical studies, translating these findings into practical clinical applications remains a critical area for future research. Particularly in the treatment of oxidative stress and lipid metabolism disorders, the clinical validation of curcumin as a potential therapeutic approach requires further investigation.

### 6.5 The future and prospects of curcumin

Chinese medicinal resources are abundant and diverse, though many remain underdeveloped and underutilized. In-depth research on individual drugs using modern methods can not only provide evidence to support traditional Chinese medicine theory but also uncover the unique therapeutic effects of these drugs on specific diseases and conditions. Additionally, exploring their modern applications can enhance the effectiveness of clinical treatments. Scientific research serves as a bridge between Traditional Chinese Medicine and modern medical theories, playing a crucial role in preserving the inheritance of TCM while also advancing its modernization. To fully utilize the potential of curcumin, future research can focus on the following aspects: first, developing novel curcumin derivatives or agents to improve its bioavailability and targeting; second, conducting large-scale, multi-center clinical trials to validate the efficacy and safety of curcumin in different disease models; third, exploring the molecular mechanisms of curcumin’s action, especially the role of crosstalk and regulation in the complex network of multiple diseases; and fourth, studying the combined application of curcumin with other drugs or lifestyle interventions to optimize therapeutic effects.

In conclusion, curcumin, a promising natural metabolite, has shown significant potential in reducing oxidative stress and regulating lipid metabolism disorders. As research advances, curcumin is likely to emerge as a key tool in the prevention and treatment of related diseases in the future.
